# Exploring the imaging features and treatment options of Mirizzi syndrome

**DOI:** 10.1016/j.radcr.2023.01.102

**Published:** 2023-03-09

**Authors:** Razi Haq, Bradley Chatterton, Lyle Gesner

**Affiliations:** Cooperman Barnabas Medical Center, Rutgers RWJ Barnabas Health, 94 Old Short Hills Rd, Livingston, NJ 07039 USA

**Keywords:** Mirizzi, Cholelithiasis, Gallstone, Common hepatic duct, Cystic duct, Common bile duct

## Abstract

The patient is a 54-year-old female who presented to the emergency department for episodic right biliary colic with nausea and vomiting over the past year. The patient's symptoms warranted multiple emergency department visits, but were self-limiting. During the most recent visit, the patient had a low-grade fever of 99.8°F (96.8°F-99.5°F) and a borderline elevated total bilirubin of 1.2 (0.2-1.2 mg/dL). Abdominal ultrasound revealed cholelithiasis, gallbladder wall thickening, and biliary ductal dilatation. Subsequent MRCP revealed an impacted stone within the gallbladder neck and a prominent common hepatic duct, compatible with Mirizzi syndrome Type I. The obtained imaging combined with clinical correlation in the setting of jaundice and right upper quadrant pain guided the patient's management. A laparoscopic cholecystectomy was performed and the patient was safely discharged the following day.

## Introduction

Mirizzi syndrome is defined as common hepatic duct obstruction as a result of stone impaction within the cystic duct or neck of the gallbladder [1,2]. Stone impaction in either location may result in a mechanical obstruction of the common hepatic duct, resulting in jaundice, fever and right upper quadrant pain [1]. These symptoms resemble other biliary pathologies and require a full workup including diagnostic imaging to determine the underlying pathology.

## Method

Observational case report.

## Case presentation

The patient is a 54-year-old female who presented to the emergency department for evaluation of episodic right upper quadrant abdominal pain over the past year. The patient described a sharp, non-radiating post-prandial pain accompanied by intermittent nausea and vomiting, for which she presented to the emergency department multiple times. These episodes were self-resolving until the most recent admission. In the emergency department, the patient had a low-grade fever of 99.8°F (96.8°F-99.5°F) and a borderline elevated total bilirubin of 1.2 (0.2-1.2 mg/dL).

**Fig. 1:**
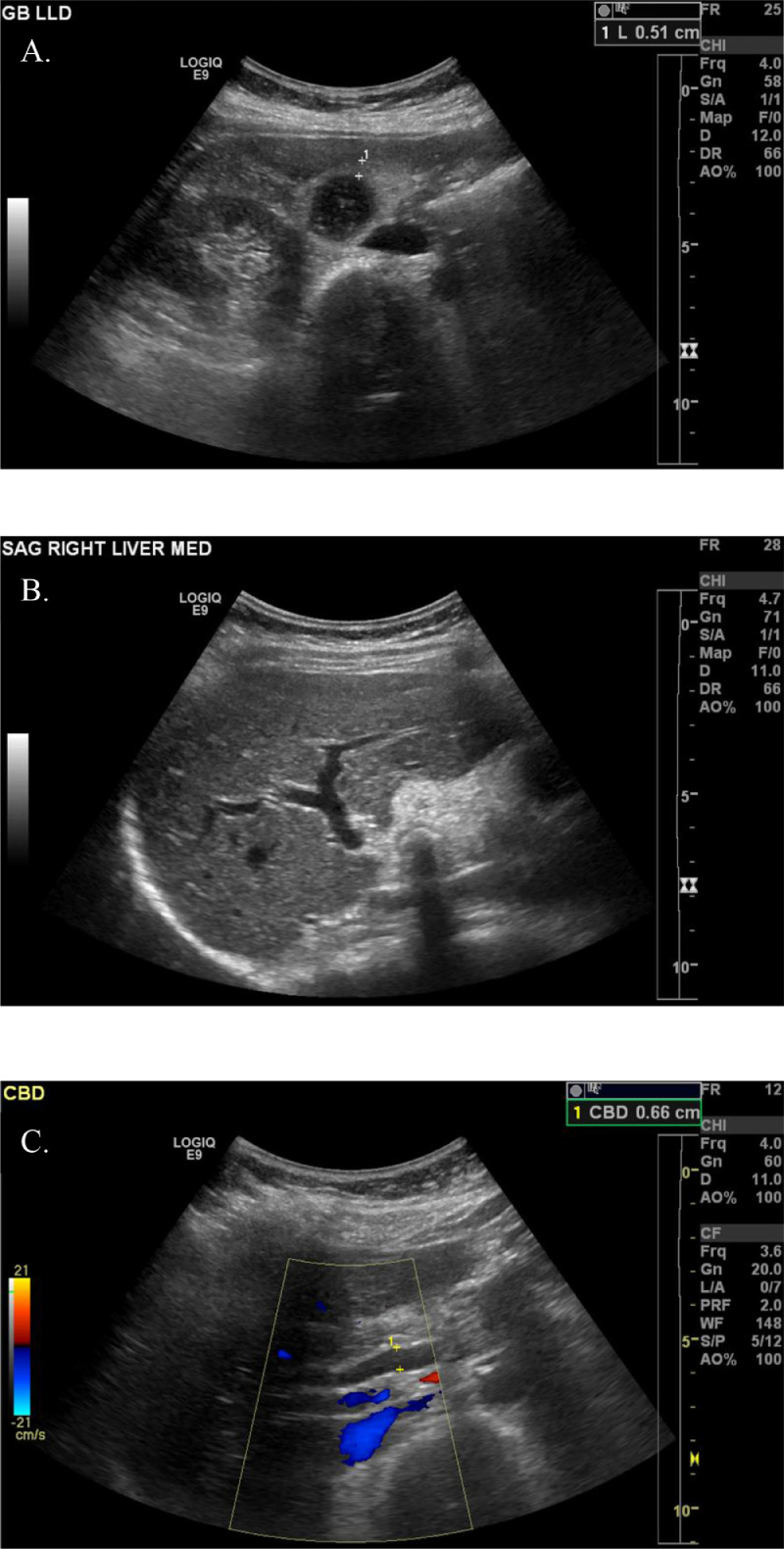
Abdominal ultrasound. Abdominal ultrasound revealing (A) thickened gallbladder with cholelithiasis, (B) intrahepatic biliary ductal dilation, and (C) extrahepatic biliary ductal dilation.

Ultrasound of the abdomen revealed a distended, thickened gallbladder, cholelithiasis, and intrahepatic and extrahepatic biliary ductal dilatation (Fig. 1).

**Fig. 2:**
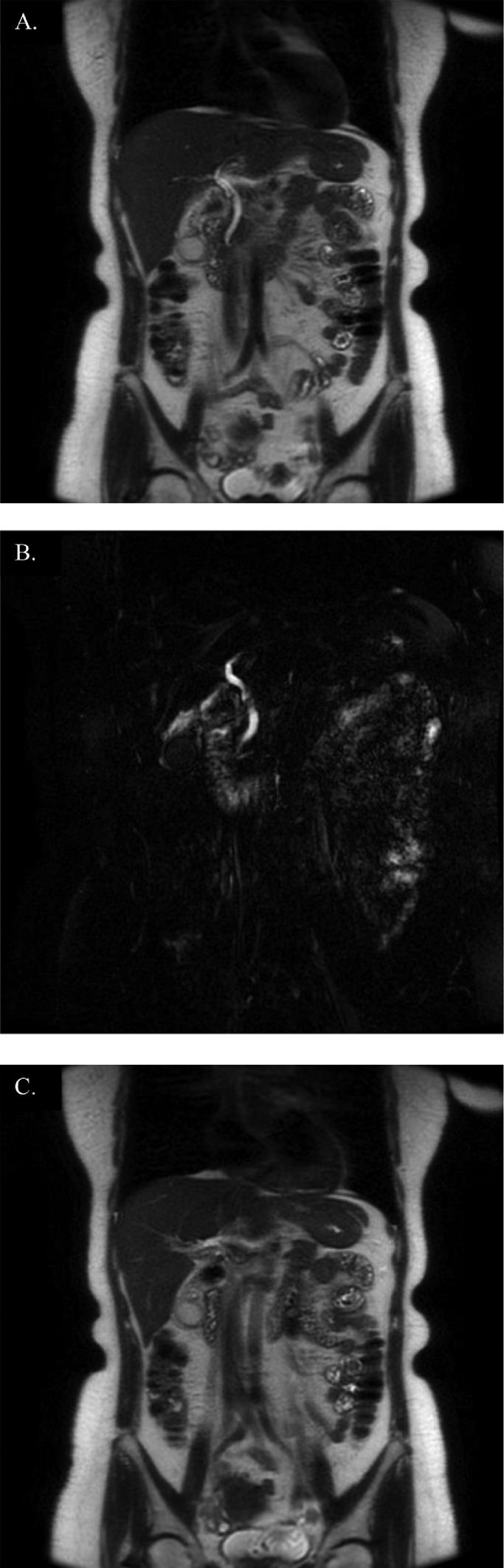
MRCP. MRCP revealing (A) and (B) impacted gallstones with mild mass effect and resultant prominence of the extrahepatic bile duct and (C) 2.2 × 1.1 × 1.0 cm impacted stone within the gallbladder neck.

Subsequent magnetic resonance cholangiopancreatography (MRCP) revealed a 2.2 × 1.1 × 1.0 cm impacted stone within the gallbladder neck with intrahepatic and extrahepatic biliary ductal prominence compatible with Mirizzi syndrome (Fig. 2).

The patient underwent laparoscopic cholecystectomy which revealed a chronically inflamed gallbladder with cholelithiasis. The patient was safely discharged the following day.

## Discussion

Mirizzi syndrome is defined as common hepatic duct obstruction secondary to gallstone impaction in the cystic duct or infundibulum of the gallbladder [1,2]. Stone impaction within the cystic duct may produce mass effect/obstruction of the adjacent common hepatic duct; thereby, producing the clinical constellation of jaundice, fever, and right upper quadrant pain [1].

Pain is the most common symptom, present in 54%-100% of cases, followed by jaundice which is present in 24%-100% of cases [3]. Mirizzi syndrome more commonly occurs in females at approximately 50%-77% [4,5]. This may be due in part to the higher incidence of gallstones in females. There is also an association between Mirizzi syndrome and gallbladder cancer [4,6]. A retrospective study found that there was an increased incidence of gallbladder cancer in patients with Mirizzi syndrome than in patients with uncomplicated gallstone disease [6].

Mirizzi syndrome can be divided into 5 types. The classification system is based on the presence as well as the extent of a cholecystobiliary fistula [7,8].

1. Type I is defined as the external compression of the bile duct by an impacted gallstone in the gallbladder infundibulum or cystic duct. Type I can be further separated into:
  a. Type IA (presence of cystic duct) and,
  b. Type IB (obliteration of the cystic duct) [9].
2. Type II is defined as a cholecystobiliary fistula secondary to erosion from the bile duct wall by a gallstone and must involve up to one-third of the bile duct circumference.
3. Type III is defined as a cholecystobiliary fistula involving up to two-third of the bile duct circumference.
4. Type IV is defined as a cholecystobiliary fistula with complete destruction of the bile duct wall and fusion with the gallbladder, forming a single structure without a discernable plane of dissection between biliary tree structures [7].
5. Type V is defined as the presence of a cholecystoenteric fistula together with any other type of Mirizzi syndrome. Mirizzi syndrome type V can be further separated into:
  a. Type VA (cholecystoenteric fistula without gallstone ileus) and,
  b. Type VB (cholecystoenteric fistula with gallstone ileus) [8].

Evaluation of biliary colic includes right upper quadrant ultrasound [9,10], in addition to Murphy sign on physical exam, complete blood count, liver function tests, and bilirubin levels. The most common ultrasound findings in Mirizzi syndrome are an impacted stone within the gallbladder neck, dilated biliary system above the level of the gallbladder neck, and an abrupt change in width of the duct below the level of the stone.

The sensitivity of ultrasound in diagnosing Mirizzi syndrome is 23%-46% [10], as each aforementioned finding in isolation is non-specific. Mirizzi syndrome can be detected on abdominal CT with a sensitivity of 42% [11]. If clinical and imaging suspicion for biliary obstruction persists, an abdominal MRI/MRCP may provide elucidation. Combined, CT and MRI/MRCP demonstrate 96% sensitivity in the diagnosis of Mirizzi syndrome [11]. If a fistula is present, percutaneous transhepatic cholangiography or endoscopic retrograde cholangiopancreatography can be performed [10].

Therapeutic intervention for Mirizzi syndrome is dependent on whether it is diagnosed preoperatively or intraoperatively, as well as the classification type. If diagnosed preoperatively, endoscopic retrograde cholangiopancreatography can decompress the biliary system via internal stenting in patients with jaundice or cholangitis [10]. Most cases of Mirizzi syndrome are diagnosed intraoperatively; if so, a cholangiogram should be performed prior to laparoscopic or open cholecystectomy to verify biliary anatomy [8,12].

1. Treatment of Type I is a cholecystectomy, and can be performed either laparoscopic or open [8].
2. Treatment of Type II is a subtotal cholecystectomy, leaving a remnant of gallbladder wall measuring about 5 mm for closure of the fistula [8].
3. Treatment of Type III is a subtotal cholecystectomy, leaving a flap of the gallbladder (at least 1 cm) to repair the bile duct. Cases with extensive inflammation of the gallbladder wall require another procedure such as a bilioenteric anastomosis to the duodenum [8].
4. Treatment of Type IV requires a bilioenteric anastomosis, preferably a hepaticojejunostomy *Roux-en-Y*, due to extensive destruction of the bile duct wall [8].
5. Treatment of Type V differs for type VA and type VB:
  a. Mirizzi syndrome Type VA is treated with cholecystectomy and closure of the fistula.
  b. Type VB is treated by first addressing the gallstone ileus (simple enterolithotomy), and then cholecystectomy after the patient has recovered (minimum of 3 months recovery) [8].

## Conclusion

Patients presenting with right upper quadrant abdominal pain typically undergo a workup which includes physical exam, laboratory studies, and imaging. In the setting of jaundice, the goal of such a workup is to identify a biliary pathology or obstruction. In our case, MRI/MRCP identified Mirizzi syndrome Type I and guided management accordingly.

## Patient consent

The patient gave consent for the publication of this case. All identifiers were eliminated in the writing of this report.
